# A prospective registry of emergency department patients admitted with infection

**DOI:** 10.1186/1471-2334-11-27

**Published:** 2011-01-26

**Authors:** Julian M Williams, Jaimi H Greenslade, Juliet V McKenzie, Kevin H Chu, Anthony FT Brown, David Paterson, Jeffrey Lipman

**Affiliations:** 1 Department of Emergency Medicine, Royal Brisbane and Women's Hospital, Brisbane, Australia; 2 School of Medicine, University of Queensland, Brisbane, Australia; 3 Faculty of Health Sciences, University of Queensland, Brisbane, Australia

## Abstract

**Background:**

Patients with infections account for a significant proportion of Emergency Department (ED) workload, with many hospital patients admitted with severe sepsis initially investigated and resuscitated in the ED. The aim of this registry is to systematically collect quality observational clinical and microbiological data regarding emergency patients admitted with infection, in order to explore in detail the microbiological profile of these patients, and to provide the foundation for a significant programme of prospective observational studies and further clinical research.

**Methods/design:**

ED patients admitted with infection will be identified through daily review of the computerised database of ED admissions, and clinical information such as site of infection, physiological status in the ED, and components of management abstracted from patients' charts. This information will be supplemented by further data regarding results of investigations, microbiological isolates, and length of stay (LOS) from hospital electronic databases. Outcome measures will be hospital and intensive care unit (ICU) LOS, and mortality endpoints derived from a national death registry.

**Discussion:**

This database will provide substantial insights into the characteristics, microbiological profile, and outcomes of emergency patients admitted with infections. It will become the nidus for a programme of research into compliance with evidence-based guidelines, optimisation of empiric antimicrobial regimens, validation of clinical decision rules and identification of outcome determinants. The detailed observational data obtained will provide a solid baseline to inform the design of further controlled trials planned to optimise treatment and outcomes for emergency patients admitted with infections.

## Background

### Emergency department and infections

Patients with infections ranging in severity from uncomplicated cellulitis to fulminant septic shock account for a significant proportion of Emergency Department (ED) workload [1]. Furthermore, studies based in the intensive care unit (ICU) reveal that the majority of patients with severe infections are admitted after first presenting to the ED and receiving initial diagnostic workup and treatment [2]. Given the key role of the ED in diagnosis, risk stratification and initial treatment of patients with infection, it is clear that ED clinicians are ideally placed to contribute significantly to research in this area. Collecting rigorous, quality observational data in this setting will provide the foundation for a significant programme of clinical research.

### Potential uses and benefits of a prospective sepsis registry

The benefits of establishing a comprehensive and detailed prospective database on patients admitted with infection are numerous. These data can provide a baseline measurement of important cohort characteristics, spectrum of disease severity, and outcomes for various subgroups. By collecting data on the investigations and treatment initiated, compliance with established best-practice guidelines can be assessed, and changes may be quantified post implementation of quality-improvement initiatives. Comprehensive data on microbiological isolates and associated sensitivities will enable the formulation of locally-valid guidelines for empirical antimicrobial therapy.

It is only with a large, quality database that prognostic associations between variables and outcome can be sought, facilitating the validation of scoring systems and decision rules established elsewhere, and the development of locally-derived scoring systems and decision rules. A critical examination of the patterns and trends in observational data may generate hypotheses and provide the basis for further experimental trials. In many circumstances observational data provide the only evidence to guide future management (e.g. time to antibiotics in septic shock) [2], because it would be unethical to test certain hypotheses within a randomised trial design. This important role of well-designed studies using detailed, prospective, observational data in advancing our knowledge and understanding of complex clinical issues has recently been emphasised [3].

### Methodological standards for clinical registries

A clinical register is defined as a database of systematically collected, health-related information and, together with the system governing the register, is known as a registry [4].

Guidelines for the structure of this type of clinical registry exist, outlining necessary characteristics such as the collection of variables for risk adjustment, indicators to assess quality of care, and outcome data. The "Operating Principles and Technical Standards for Australian Clinical Quality Registries"[4] exhaustively specifies further desirable characteristics of clinical registries regarding the mode of data collection, data elements, risk-adjustment factors, data security and quality, organisation and governance, ethics and privacy. Our database has been designed to comply with as many of these characteristics as is practically possible.

### Review of previous studies

There are many examples of quality observational databases that contribute significantly to the knowledge and understanding of complex clinical problems and provide frameworks from which to direct scientific enquiry. These include the National Traumatic Coma Database [5] and the Australian and New Zealand Intensive Care Society Adult Patient Database [6]. Most clinical databases of patients with infections include only those with severe sepsis or septic shock [2,7] and are often multi-centred and/or ICU-based. Databases of ED patients with infections of all severities are less common, but examples do exist and are represented in numerous studies that have contributed significantly to the ED literature.

Researchers at the Beth Israel Deaconess Medical Centre in Boston have collected rigorous prospective data on emergency patients admitted with infection over several discrete time periods. Using the ordering of blood cultures by the treating emergency physician as an indicator of suspected infection, data were collected on a series of 3,179 patients between February 2000 and February 2001. A number of studies have since been published between 2003 and 2010 using this dataset, including a paper describing the derivation of the Mortality in Emergency Department Sepsis (MEDS) score [8]. This is a severity scoring system that was subsequently validated at one year [9] and was the subject of a recent complimentary review [10]. A further dataset publication provided evidence that the Charlson Co-morbidity score [11], a four-point score developed to objectively quantify the burden of co-morbid illness, also predicted one-year mortality in these patients [12]. A 2006 paper explored the prognostic implications of organ dysfunction and the presence of the Systemic Inflammatory Response Syndrome (SIRS) [13], and a recent publication found a poor association between abnormal temperature or leucocytosis and subsequently proven bacteraemia [14].

Data on several large cohorts of emergency patients admitted with clinical suspicion of infection were again collected at Beth Israel Deaconess Medical Centre between 2003 and 2006. At least seven studies have been published using these data, advancing our knowledge and understanding of emergency patients with infections and the associated determinants of outcome. Several severity scoring systems have been validated [15] and the association between lactate and poor outcome has been explored in detail [16,17]. Donnino et al. [18] concluded co-incident treatment with "statins" (hydroxy-methyl-glutaryl co-enzyme A inhibitors) was associated with improved outcomes in patients admitted with infections, and a rule comprising weighted risk factors for poor outcome in elderly patients admitted with infection was derived and validated [19]. In recently published studies, early abnormalities in coagulation system parameters were independently associated with poor outcome [20], and factors associated with clinical deterioration and transfer to ICU after admission to a ward were identified [21].

The productivity of the investigators at Beth Israel Deaconess Medical Centre summarised above demonstrates that the creation and maintenance of a large database of detailed information on emergency patients with the full spectrum of disease severity is achievable and can contribute substantially to our understanding of this complex disease process and its management. Large observational databases should ideally be multi-centred, national or even international in scope in order to maximise power and generalisability [4]. However, the labour-intensive process of data abstraction from paper ED records makes a single-centre registry a more practical proposition. The inclusion of large numbers of patients, admitted with all severities of infection, will enhance the power of observations made and the relevance of these observations to emergency medicine clinicians and researchers.

### Objective

The objective of this paper is to describe the creation of a comprehensive, systematic and detailed database of the way in which patients with infections present and are treated at a typical inner city university hospital ED. Data regarding microbiological isolates and their associated sensitivities will enable the optimisation of empirical guidelines for antimicrobial therapy. Together with accurate outcome data, it will be possible to assess compliance with established guidelines and best practice, validate scoring systems, investigate factors of prognostic significance, and provide solid baseline data to inform further studies and quality improvement initiatives.

## Methods/Design

### Setting and study population

This study will be conducted in the ED of an adult inner city university hospital in Australia with an annual census of over 72,000. Patients eligible for inclusion will be those presenting to the ED and subsequently admitted to hospital with a primary diagnosis indicating clinical suspicion of infection. Those patients transferred from another hospital or under the age of 18 years will be excluded.

### Data collection process

On a daily basis, trained data abstractors will scrutinise the hospital electronic database record of emergency patients admitted over the 24-hour period to midnight the night before. Those patients with an admission diagnosis indicating or suggestive of infection according to International Classification of Diseases version 10 coding will be listed (see additional file 1), and the charts of the patients thus identified will be examined to assess suitability for study inclusion. Only those patients that are judged to have infection as the most likely cause for their admission according to both the treating ED senior medical officer and the admitting team will be enrolled.

Data will be abstracted from the charts of enrolled patients, with information recorded on case report forms and subsequently entered into a secure computerised study database (Access, Microsoft, Redmond, WA). We have shown with pilot data that with appropriate training, our data abstractors achieve a high degree of inter-rater agreement (97%). At a later date this clinical information will be supplemented by further information from other hospital electronic databases, regarding pathology and microbiology results and hospital length of stay (LOS). Mortality outcome data will be obtained from periodic interrogation of the Australian Institute of Health and Welfare National death index [22]. This registry records vital status data for all individuals deceased within Australia.

### Variables recorded

Table 1 lists the variables recorded in the database. Clinical details such as site of infection, physiological status and treatment in ED and co-morbid conditions will be abstracted directly from the hospital paper chart. Values recorded for physiological status will be the most abnormal values recorded in the ED, and missing physiological values will be recorded as such and assumed to be in the normal range for analysis purposes. Results of laboratory tests and microbiological assays will be transferred manually from hospital electronic databases.

**Table 1:** Variables recorded in the database.

Data type	Variables recorded
Demographics	Age, gender, postcode

Site of infection	Classified: respiratory, urinary tract, abdominal/pelvic, soft tissue, skeletal, neurological, vascular, unknown source.

Aetiological agents identified	Results of positive cultures, serology, antigen tests, nucleic acid amplification.

Physiological status in ED	SIRS criteria, initial and lowest systolic blood pressure, oliguria (urine output less than 0.5 ml/kg/hour for 2 consecutive hours), oximetry values.

Pathology tests in ED	Results of biochemistry and haematology profiles. Further data may also be available such as results of blood gas analyses and coagulation profiles.

Treatment in ED	Amount and type of fluid administered Type and timing of antimicrobial therapy

Other variables	Limitations on therapy (e.g. not for ICU admission) Nursing home status Antibiotics prior to admission Hospital admission in the past month

Severity of illness	MEDS score SAPS II score

Co-morbid illness	Charlson Co-morbidity score

Organ dysfunction	Modified SOFA score

Outcomes	Hospital length of stay ICU length of stay (where applicable) Date of death (where applicable)

MEDS = Mortality in Emergency Department Sepsis; SAPS II = New Simplified Acute Physiology Score; SOFA = Sequential Organ Function Assessment.

Microbiological isolates cultured from samples taken in the ED and up to 48 hours after presentation will be deemed potentially relevant causative organisms. A more liberal approach will be taken regarding results from other tests such as serology, antigen tests and nucleic acid amplification assays. Cases in which clinical interpretation is required in order to clarify issues such as site of infection and relevance of microbiological isolates will be referred to a panel comprising an Emergency Physician, Intensivist, and Infectious Disease Physician/Microbiologist for adjudication.

### Derived variables

A number of derived variables or scores will also be recorded in the database. The Mortality in Emergency Department Sepsis (MEDS) score sums nine weighted components: (age > 65 years, terminal illness, tachypnoea/hypoxia, shock, thrombocytopaenia, bands >5%, altered mental status, nursing home residence, and lower respiratory tract infection) and has been validated as an accurate mortality-risk stratification tool in ED patients with infection [15,23]. Our laboratory does not routinely measure leukocyte bands, so effectively a modified MEDS score comprising the remaining eight components will be recorded.

The Simplified Acute Physiology Score [24] was originally developed as a predictive tool in intensive care patients and has subsequently been shown to have utility in ED patients [25,26]. This score is a function of 17 weighted clinical variables and estimates the probability of hospital mortality. The Charlson Co-morbidity score is a frequently-used index of co-morbid illness burden, which has been validated in a similar cohort of emergency patients with infection [12]. The calculation of these three scores will enable score validation in our cohort, allow comparison of our patients with those in other studies, and facilitate adjustment for severity of illness and burden of co-morbidity in regression analyses.

### Indices of Organ Dysfunction

A modified SOFA score [27] will be used to indicate and quantify organ dysfunction across six organ systems (see table 2). The SOFA score is a validated and widely used tool for quantifying organ dysfunction in patients with infection, and is a preferred method listed by the Surviving Sepsis Campaign consensus guidelines [28]. Several modifications to the score as originally published have been made for the purposes of this study. The published SOFA cardiovascular system (CVS) score incorporates the potential use of a variety of vasopressor and inotropic agents, including noradrenaline, adrenaline, dopamine and dobutamine. The modified SOFA cardiovascular system score reflects local practice of using noradrenaline almost exclusively as the vasopressor of choice in patients with septic shock. Adrenaline will be considered equivalent to noradrenaline for the purposes of calculating the cardiovascular system score, and vasopressor infusion must be required for at least one hour.

**Table 2:** modified SOFA score.

		Modified SOFA score				
Organ system	Determinants	0	1	2	3	4

RESP	PaO_2_/FiO_2 _+ SaO_2_%	>400 >94% (RA)	<400 90-94%	<300 <90% (RA)	<200	<100

CVS	Blood pressure, + Vasopressor requirement	SBP > 90 at all times	SBP > 90 only after fluid bolus 20-30 ml/kg	SBP < 90 despite fluid bolus. NA < 8 (mcg/min)	NA 8-15 (mcg/min)	NA > 15 (mcg/min)

HAEM	Platelet count (x10^9^/l)	>150	<150	<100	<50	<20

GIT	Bilirubin (micromol/l)	<20	20-32	33-101	102-204	>204

CNS	Glasgow Coma Scale (GCS) score	15	13-14	10-12	6-9	3-5

RENAL	Creatinine (micromol/l) + Urine output (ml/kg/hour)	<120	>120 Or UO <0.5 for 2 hours	>170	>300	>440

RESP = respiratory system; CVS = cardiovascular system; HAEM = haematological system; GIT = gastro-intestinal system; CNS = central nervous system; RENAL = renal system;

PaO_2 _= arterial partial pressure of oxygen (mmHg); FiO_2 _= fractional inspired oxygen; RA = room air; SBP = systolic blood pressure (mmHg); NA = noradrenaline; GCS = Glasgow Coma Scale; UO = urine output.

Our respiratory system score utilises the ratio of arterial partial pressure of oxygen to fractional inspired oxygen (PaO_2_/FiO_2_) as originally published, with the addition of pulse oximetry saturation (SpO_2_) >94% on room air indicating no respiratory dysfunction, and SpO_2_<90% on room air indicating a respiratory SOFA score of two. These additions were made to reflect the fact that many ED patients will not have an arterial blood gases analysis performed, but SpO_2 _readings will be obtained in almost all patients.

Lastly, creatinine concentration cut-offs have been slightly adjusted at the lower scores to reflect the local laboratory reference range, and oliguria (defined as urine output less than 0.5 ml/kg/hour for two consecutive hours) scoring two points has replaced an impractical series of daily urine balances as detailed in the original SOFA score. The other organ system SOFA scores (haematology, central nervous system, and gastrointestinal) remain unchanged from the original SOFA score gradings.

The proposed dataset includes a comprehensive description of all relevant characteristics of patients with infections that are routinely measured at the time of ED assessment. The recording of SIRS criteria and SOFA scores enables stratification of database patients into the categories of sepsis, severe sepsis and septic shock as defined by the Surviving Sepsis Campaign consensus guidelines [28]. The collection of sufficient data to calculate MEDS and SAPS II scores for each patient will facilitate validation of these scores, and comparison of patients in studies using this database with other patient cohorts.

### Data integrity

A number of strategies will be introduced to ensure the quality of the data entered in the registry:

1. All individuals collecting the data for the registry will be trained in data collection and data entry methods.

2. The database incorporates validation fields which provide warnings if a data value falls outside the expected range.

3. The database will be checked on a monthly basis for completeness. A report of missing data will be printed and provided to the data collection team for follow-up.

4. A data audit will be conducted every six months. Within this audit, the following data checks will be conducted:

a. Every field will be checked for values outside the standard reference range. Extreme values will be investigated for accuracy.

b. All data will be rechecked against original paper-based data.

### Data security

The registry is a Microsoft Access document that is stored on a protected network drive, which is accessible only to authorised users. Individuals with access to the network drive must enter a second password to allow them entry to the database. There are two levels of access to the database.

1) User access. This access is available to data collectors and only allows data entry.

2) Super-user access. This access enables users to view and modify all sections of the database.

The database contains a separate file that links the patient's study ID number to their hospital identification number, which will enable re-identification of the data if necessary. This file is accessible only to super-users and requires a separate password to be opened.

### Ethics approval

The collection of this data as described above has been approved by the Royal Brisbane and Women's Hospital Human Research Ethics Committee (HREC). A waiver for informed consent was obtained owing to the entirely observational nature of the project. Ethics approval will be sought for further studies conducted using the database.

### Limitations

Our reliance on the diagnosis field of the ED admissions database to identify potential study candidates may result in some eligible patients admitted with infection being missed. It is possible to enter in the ED diagnosis field a major symptom such as abdominal pain or headache, rather than an established diagnosis. During data collection, an attempt will be made to list and peruse the charts of not only those patients with clear diagnoses indicating infection, but also those admitted with symptoms that may be suggestive of infection.

It is also possible that patients may be included but ultimately discharged with a diagnosis other than infection. Inadvertent inclusions will be minimised by enrolling only those patients with both ED and admitting team diagnosis indicating infection.

The acquisition of data from patients' charts has the potential to result in incomplete or incorrect data, especially regarding relevant co-morbid conditions. However the abstraction of data with access to patients' full hospital records as planned will minimise this effect.

## Discussion

This database will provide substantial insights into the characteristics, microbiological profile, and outcomes of emergency patients admitted with infections. It will provide the substrate for a programme of research into compliance with evidence-based guidelines, the validation of clinical decision rules and the identification of outcome determinants.

The detailed observational data obtained will provide solid baseline data with which to modify empiric antimicrobial regimes and to inform the design of further controlled trials planned to optimise treatment and outcomes for emergency patients admitted with infections.

## Competing interests

The authors declare that they have no competing interests.

## Authors' contributions

JW, JM and JG conceived, designed and refined the data collection process and database structure. AB and KC contributed to the text and strategic guidance was provided by JL and DP. All investigators read and approved the final manuscript.

## Pre-publication history

The pre-publication history for this paper can be accessed here:

http://www.biomedcentral.com/1471-2334/11/27/prepub

## Supplementary Material

Additional file 1 **ED admission ICD-10 codes potentially indicating infection**. Click here for file
